# Wearable Electronics and Smart Textiles: A Critical Review

**DOI:** 10.3390/s140711957

**Published:** 2014-07-07

**Authors:** Matteo Stoppa, Alessandro Chiolerio

**Affiliations:** Center for Space Human Robotics, Istituto Italiano di Tecnologia, Corso Trento 21, 10129 Torino, Italy

**Keywords:** smart textiles, electronic textiles, wearable systems

## Abstract

Electronic Textiles (e-textiles) are fabrics that feature electronics and interconnections woven into them, presenting physical flexibility and typical size that cannot be achieved with other existing electronic manufacturing techniques. Components and interconnections are intrinsic to the fabric and thus are less visible and not susceptible of becoming tangled or snagged by surrounding objects. E-textiles can also more easily adapt to fast changes in the computational and sensing requirements of any specific application, this one representing a useful feature for power management and context awareness. The vision behind wearable computing foresees future electronic systems to be an integral part of our everyday outfits. Such electronic devices have to meet special requirements concerning wearability. Wearable systems will be characterized by their ability to automatically recognize the activity and the behavioral status of their own user as well as of the situation around her/him, and to use this information to adjust the systems' configuration and functionality. This review focuses on recent advances in the field of Smart Textiles and pays particular attention to the materials and their manufacturing process. Each technique shows advantages and disadvantages and our aim is to highlight a possible trade-off between flexibility, ergonomics, low power consumption, integration and eventually autonomy.

## 1. Introduction

The term “Smart Textiles” refers to a broad field of studies and products that extend the functionality and usefulness of common fabrics. Smart Textiles are defined as textile products such as fibers and filaments, yarns together with woven, knitted or non-woven structures, which can interact with the environment/user. The convergence of textiles and electronics (e-textiles) can be relevant for the development of smart materials that are capable of accomplishing a wide spectrum of functions, found in rigid and non-flexible electronic products nowadays. Smart Textiles will serve as a means of increasing social welfare and they might lead to important savings on welfare budget. They integrate a high level of intelligence and can be divided into three subgroups:

- Passive smart textiles: only able to sense the environment/user, based on sensors;
- Active smart textiles: reactive sensing to stimuli from the environment, integrating an actuator function and a sensing device;
- Very smart textiles: able to sense, react and adapt their behavior to the given circumstances.

Sensors provide a nervous system to detect signals, thus in a passive smart material, the existence of sensors is essential. The actuators act upon the detected signal either autonomously or from a central control unit [1]; together with the sensors, they are the essential element for active smart materials. Fabric-based sensing has been a large field of research in the biomedical and safety communities [2]. The fabric sensors can be used for electrocardiogram (ECG) [3], electromyography (EMG) [4], and electroencephalography (EEG) [5,6] sensing; fabrics incorporating thermocouples can be used for sensing temperature [7]; luminescent elements integrated in fabrics could be used for biophotonic sensing [8]; shape-sensitive fabrics can sense movement, and can be combined with EMG sensing to derive muscle fitness [9]. Carbon electrodes integrated into fabrics can be used to detect specific environmental or biomedical features such as oxygen, salinity, moisture, or contaminants [10,11].

Active functionality could include power generation or storage [12], human interface elements [13], radio frequency (RF) functionality, or assistive technology [14]. All electronic devices require power, and this is a significant design challenge for Smart Fabrics. Power generation can be achieved through piezoelectric [15] elements that harvest energy from motion or photovoltaic elements [16]. Human interfaces to active systems can be roughly grouped into two categories: input devices and annunciation or display devices. Input devices can include capacitive patches that function as pushbuttons [17], or shape-sensitive fabrics [18] that can record motion or flexing, pressure, and stretching or compression. Annunciation and display devices may include fabric speakers [17], electroluminescent yarns [19], or yarns that are processed to contain arrays of organic light emitting diodes (OLEDs) [20]. Fabrics can also include elements that provide bio-feedback [21] or simply vibrate. Fabric-based antennas are a relatively simple application of Smart Fabrics. Simple fabric antennas are merely conductive yarns of specific lengths that can be stitched or woven into non-conducting fabrics [22].

**Figure 1.**
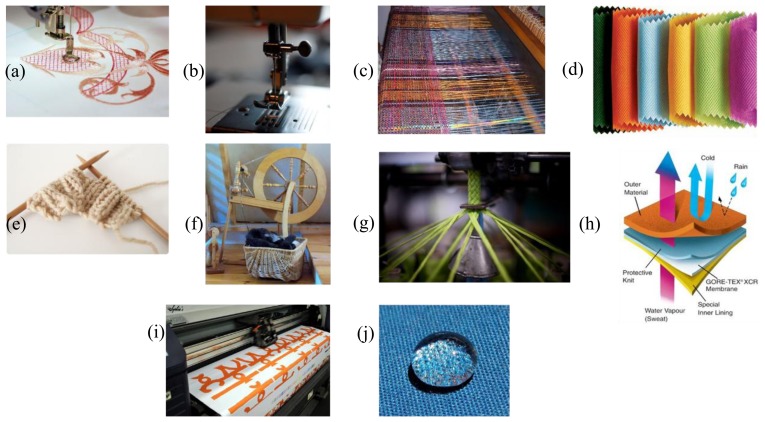
Different kinds of textile/fabric manufacturing and treatment. (**a**) Embroidery; (**b**) sewing; (**c**) weaving; (**d**) non-woven; (**e**) knitting; (**f**) spinning; (**g**) breading; (**h**) coating/laminating; (**i**) printing and (**j**) chemical treatment.

A study about intelligent textiles is at his first stage reduced to a study on smart materials. In a second phase, it is to be considered in which way these smart materials can be processed into a textile material. These smart materials are incorporated into the textile structure by different technologies (Figure 1). Among those we may list embroidering [23], sewing, non-woven textile, knitting [24], weaving [25], making a spinning [26], braiding [27], coating/laminating [28], printing [29] and chemical treatments [30] that provide specific features such as controlled hydrophobic behavior.

Innumerable combinations of these source materials result into a whole range of textiles but sometimes the commercial output is represented by garments that contain conventional cables, miniaturized electronic components and special connectors. As humans prefer to wear comfortable textiles rather than hard, rigid boxes, first efforts have been made to use the textiles themselves for electronic functions [31].

Smart Textiles present a challenge in several fields such as the medical, sport, and artistic communities, the military and aerospace. The early European Commission's 6th and 7th framework programs provided significant research and development funding for personal health monitoring through smart wearable systems and for projects targeting the integration of sensors, energy sources, processing, and communication inside the clothing. The list below shows the projects funded by the European Commission's 6th and 7th Framework programs that have focused on smart fabrics and interactive textiles (Table 1) [32,33].

Particular attention in this review is devoted to describing the materials and methodologies to develop smart textiles. Each scientific approach will be followed by a review of the related work carried out by companies, universities or research institutes.

## 2. Fabrication Techniques

Over the past decade, many techniques and materials have been used in order to realize *smart textiles*. In the following section, together with metodologies, the relative projects are also presented.

### 2.1. Conductive Fibers

Initially, conductive threads were mainly used in technical areas: clean room garments, military apparel, medical application and electronics manufacturing [34]. Textile structures that exhibit conductivity or serve an electronic or computational function are called electro-textiles [35]. They can have a variety of functions, like antistatic applications [36], electromagnetic interference shielding (EMI) [37], electronic applications, infrared absorption or protective clothing in explosive areas [38].

**Figure 2.**
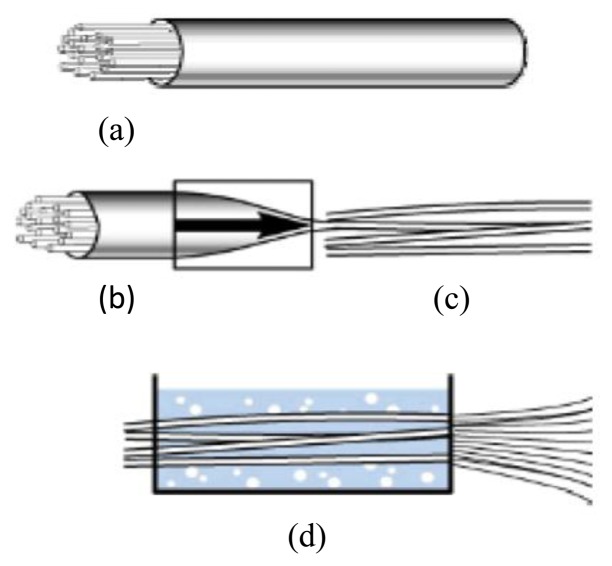
(**a**) Metal coated wire combined in iron tube; (**b**) Several diameter reductions of tube; (**c**) Bundling of tubes; (**d**) Leaching, realizing fibers.

The conventional process to produce metal fibers is wire drawing, a mechanical production process. This process is characterized by its various drawing steps, called coarse, medium, fine and carding train (Figure 2).

The drawing die, used to draw the fiber, consists of a steel mount with a core out of ceramics, carbide or diamond. The initial diameter of the metal wire varies depending on the material. For copper, for instance, it is usually is 8 mm, while for iron it is 5 mm. After drawing, the wire is annealed at temperatures ranging between 600 and 900 °C. Subsequently, they are quenched. The fine metal wire is then wrapped onto a revolving wire drawing cylinder [39].

#### Related Works

The Swiss company Elektrisola Feindraht AG (Escholzmatt, Switzerland) produces metal monofilaments that can be blended with all sorts of fibers or that can be directly used in weaving and knitting. Importantly, according to the material used, there are different electrical properties (see Table 2). The products range from copper (Cu) and silver-plated copper (Cu/Ag) filaments, brass (Ms) and silver-plated brass (Ms/Ag) filaments, aluminum (Al) filaments to copper-clad aluminum (CCA) filaments [40].

**Figure 3.**
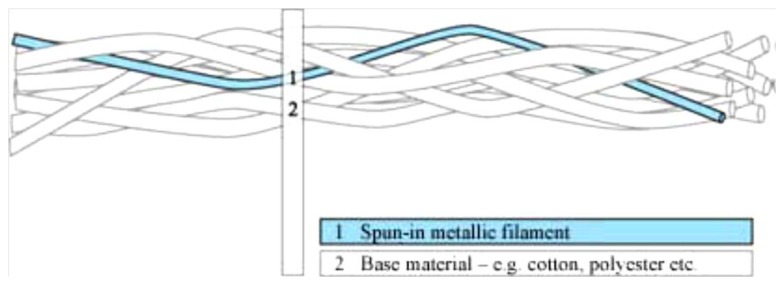
Schematic of conductive fiber twisted with the normal fibers.

The company Swiss-Shield^®^ (Flums, Switzerland) specializes in producing metal monofilaments which are incorporated into base yarns like cotton, polyester, polyamides and aramides. The metal monofilaments are made out of copper, brass, bronze, silver, gold, aluminum, for instance. The following Figure 3 shows a typical conductive yarn with base fibers and a metal monofilament twisted around them [41].

### 2.2. Treated Conductive Fibers

Instead of attaching electronics to textile substrates, the yarns of the textile can be functionalized with electronics. Electrically conductive fibers can also be produced by coating the fibers with metals, galvanic substances or metallic salts. Coatings can be applied to the surface of fibers, yarns, or even fabrics to create electrically conductive textiles. Common textile coating processes include electroless plating, evaporative deposition, sputtering, coating the textile with a conductive polymer [42].

In [43] a method to fabricate fibers with different material layers and material structuring is presented. The fabrication process is based on the conventional preform-based fiber-processing, easily yielding kilometers of functional fiber during the process.

**Figure 4.**
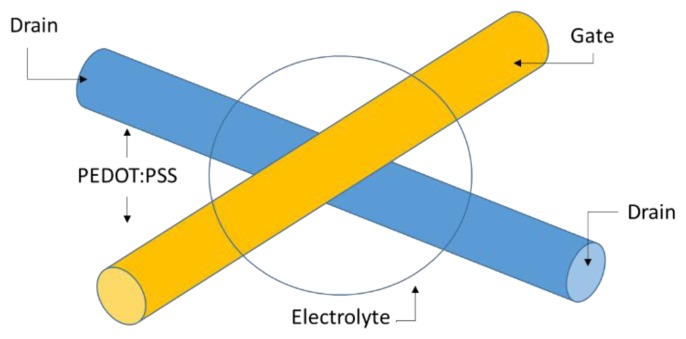
Yarn-based transistor.

Another relevant work is to use the crossing yarns in a textile to fabricate a transistor [44,45]. A schematic of a yarn-based transistor is shown in Figure 4. The resulting transistor shows an on-off current ratio of more than 1000, operated with a gate voltage of 1.5 V.

Figure 3 represents two yarns coated with PEDOT:PSS, one serving as the gate contact for the transistor while the second serves as drain and source contact. At the crossing of the two yarns, an electrolyte is placed. A redox process at the interface between electrolyte and PEDOT:PSS turns the transistor on and off [44].

#### Related Works

The Textile Research Institute of Thuringia-Vogtland (TITV, Greiz, Germany) has succeeded in producing conductive threads by coating a conventional yarn with metal layers, called ELITEX^®^. They used Shieldex Nylon 66 threads that are coated with a thin silver layer as base material. With a specific conductivity of about 1.2 × 10^3^ S·cm^−1^, the threads have a specific resistance of about 8.34 O·mm^2^/m. Hence, the resistivity is too high to conduct current [46].

### 2.3. Conductive Fabrics

**Figure 5.**
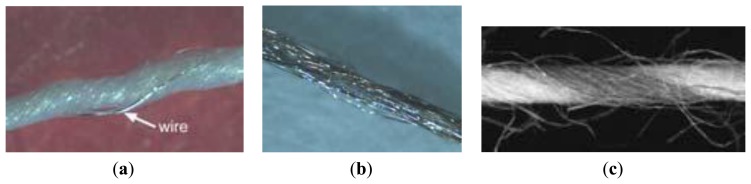
(**a**) Twisted metal wire: The metal wire is twisted around the polymer yarn; (**b**) Metal coating: The polymer yarn is physically/chemically coated with a thin metal layer; (**c**) Metal fibers: The conductive yarn consists of metal multifilaments [47].

There are different ways to produce electrically conductive fabrics. One method is to integrate conductive yarns in a textile structure, e.g., by weaving. However, the integration of conductive yarns in a structure is a complex and seldom a uniform process as it needs to be ensured that the electrically conductive fabric is comfortable to wear or soft in touch rather than hard and rigid. Conductivity can be established with different thread types (Figure 5):

However, woven fabric structures can provide a complex network that can be used as elaborated electrical circuits with numerous electrically conducting and non-conducting constituents, and be structured to have multiple layers and spaces to accommodate electronic devices.

**Figure 6.**
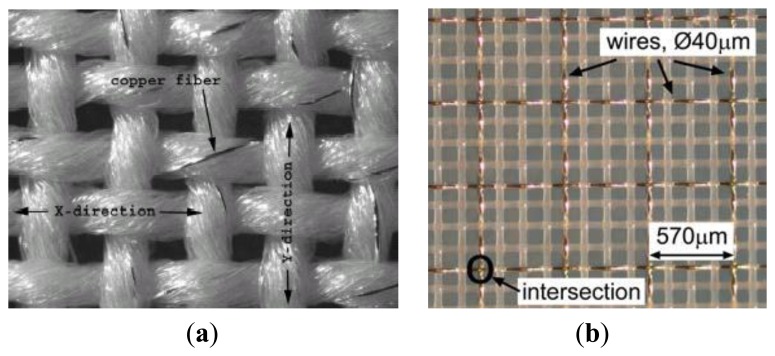
(**a**) Standard design of copper yarn twisted with polyester fibers; (**b**) PETEX.

Researchers at the Electronics Department and the Wearable Computing Laboratory at the ETH in Zürich produced a plain woven textile structure consisting of polyester yarns that are twisted with one copper thread. Initially, they started with a standard design (Figure 6a), then the researchers design a hybrid fabric called PETEX (Figure 6b) [47]. It consists of woven polyester monofilament yarn (PET) with diameter of 42 μm and copper alloy wires with diameter 50 ± 8 μm (AWG 461). Each copper wire itself is coated with a polyurethane varnish as electrical insulation. The copper wire grid in the textile features a spacing of 570 μm (mesh count in warp and in weft is 17.5 cm^−1^).

**Figure 7.**
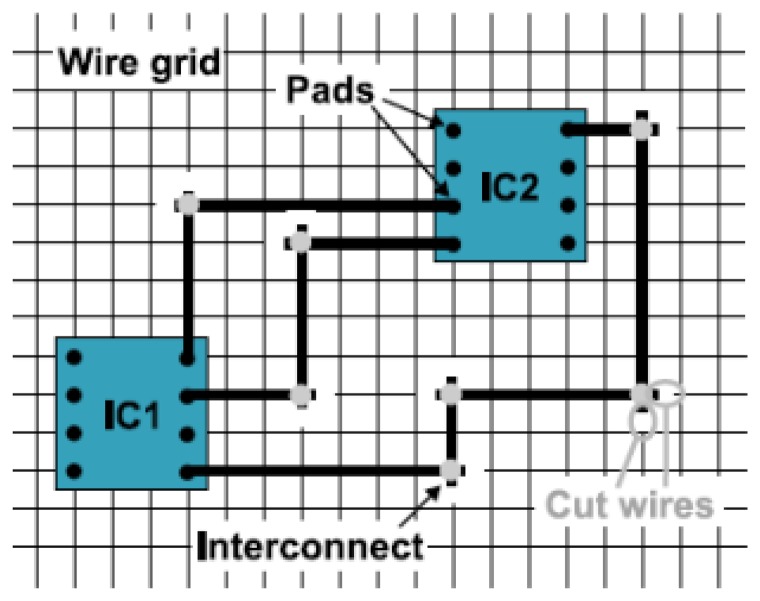
Approach to integrate circuits in a fabric with wire grid.

With the PETEX the ETH researchers introduced a new approach to Smart Textiles and in particular a new manufacturing method. The aim was the possibility to realize a custom textile circuit (Figure 7). The wiring structure among circuit components is established by connecting the fabric embedded copper wires. Cuts must be placed at specific locations in the wiring in order to avoid short-circuits between copper wires. In particular, the procedure is as follows [47]:

- coating removal on copper wires at defined intersections with laser ablation;
- cutting the wires avoiding the signal leakage with laser;
- creating the interconnection with a drop of conductive adhesive;
- adding mechanical and electrical protection with an epoxy resin deposition.

#### Related Works

The British company Baltex (Ilkeston, UK) uses the knitting technology to incorporate metal wires in textile structures. Their fabrics, which they commercialize under the name Feratec^®^, can be used mainly for two purposes, namely heatable textiles and electro-magnetic shielding materials [48].

The American company Thremshield LLC (Niagara Falls, NY, USA) produces metallized woven nylon fabrics in different shapes and profiles. The metals they use are silver, copper or a combination of copper and nickel [49].

The Danish company Chr. Dalsgaard Project Development (Aarhus, Denmark) works with the development of weaving electronics into fabrics, electronic conductors in clothing, operating panels in textiles (soft keyboards, displays, *etc.*) and micro-sensors. The conductive yarn they use is a copper thread, plated with a silver layer and coated with polyester [50].

**Figure 8.**
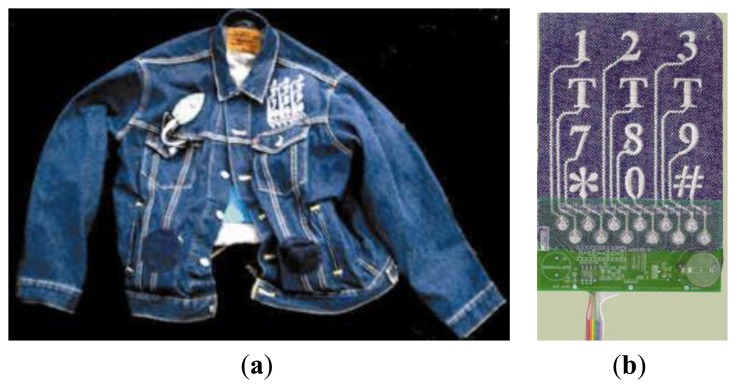
(**a**) Musical Jacket comprising a fabric keypad on one side, a MIDI synthesizer on the other side, speakers behind speaker grills in the pockets and fabric buses visible inside the jacket; (**b**) The fabric keypad with the circuit board placed behind it.

Another possibility to achieve a conductive fabric is to attach a conductive structure to a ground structure by using the embroidery technique. In 2000, the Massachusetts Institute of Technology Media Laboratory researches were the first to propose a way of stitching patterns that can define circuit traces, component connection pads, or sensing surfaces designed with traditional CAD tools for circuit layout (Figure 8) [31].

### 2.4. Conductive Inks

Interactive electronic textiles can also be produced by using conductive inks. First of all conductive inks must contain an appropriate highly conductive metal precursor such as Ag, Cu, and Au NPs and a carrier vehicle. Most of them are water based: water is the main ink component and to limit contaminants, it must be as pure as possible. These specialized inks can be printed onto various materials, among them textiles, to create electrically active patterns. Screen printing also makes integration with planar electronics simpler than with conductive yarn systems.

There are several technologies that can print conductive material on different substrate. Sheet-based inkjet and screen printing are best for low-volume, high-precision work.

Inkjets are flexible and versatile, and can be set up with relatively low effort [51]. Inkjets offer lower throughput of around 100 m^2^/h and lower resolution (*ca.* 50 μm). It is well suited for low-viscosity, soluble materials like organic semiconductors. With high-viscosity materials, like organic dielectrics, and dispersed particles, like inorganic metal inks, difficulties due to nozzle clogging occur. Because ink is deposited via droplets, thickness and dispersion homogeneity is reduced. Simultaneously using many nozzles and pre-structuring the substrate allows improvements in productivity and resolution, respectively [52].

For inkjet printing, the inks should respect the following requirements [53]:

▪ high electrical conductivity;
▪ resistance to oxidation;
▪ dry out without clogging the nozzle during printing;
▪ good adhesion to the substrate;
▪ lower particle aggregation;
▪ suitable viscosity and surface tension.

Inks may also contain additives which are used to tune ink properties or to add specific properties thus increasing its performance [54].

After inkjet printing of a metal NP-based ink, in order to form a conductive printed pattern, particles must be sintered to create continuous connectivity between them and obtain electrical percolation. Sintering is the process of welding particles together at temperatures below the corresponding bulk metal melting point, involving surface diffusion phenomena rather than phase change between the solid and the liquid [55]. For instance, with inks based on gold NPs (1.5 nm diameter), the melting temperature was experimentally found to be as low as 380 °C, while for inks based on silver NPs (15 to 20 nm in diameter), a complete sintering can be obtained down to 180 °C [54,56].

Screen printing is appropriate for fabricating electrics and electronics due to its ability to produce patterned, thick layers from paste-like materials. The screen printing procedure, a stencil process, comprises the printing of a viscous paste through a patterned fabric screen and is usually followed by a drying process. The method can be applied to flat or cylindrical substrates. Depending on the substrate materials and the requirements for the printed structures, a high temperature densification can also be necessary (organic substrate T < 150 °C. Glass, ceramic and metal substrate T > 500 °C) [57].

This method can produce conducting lines from inorganic materials (e.g., for circuit boards and antennas), but also insulating and passivating layers. Generally, the throughput is about 50 m^2^/h with a resolution lower than 100 μm. By optimizing of the process condition and material the resolution may decrease to 30 microns line/space on thin flexible substrates [58].

This versatile and comparatively simple method is used mainly for conductive and dielectric layers [59], but also organic semiconductors can be printed, e.g., for OPVCs and OFETs [60].

**Figure 9.**
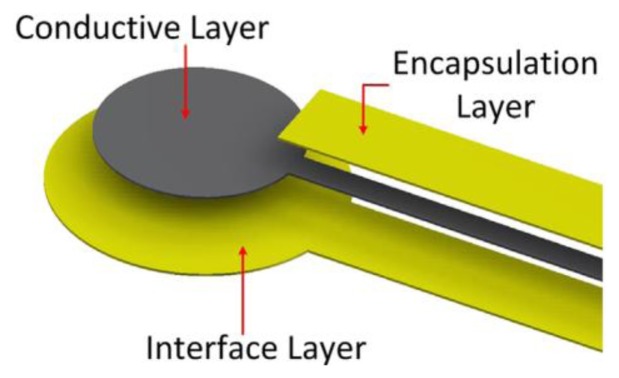
Screen printing fabrication for conductive tracks.

Recently the researchers of University of Southampton [61] have developed an innovative screen printed network of electrodes and associated conductive tracks on textiles for medical applications (Figure 9). A polyurethane paste is screen printed on to a woven textile to create a smooth, high surface energy interface layer and a silver paste is subsequently printed on top of this interface layer to provide a conductive track. Silver pastes have been printed on to non-woven textiles to create wearable health monitoring devices.

**Figure 10.**
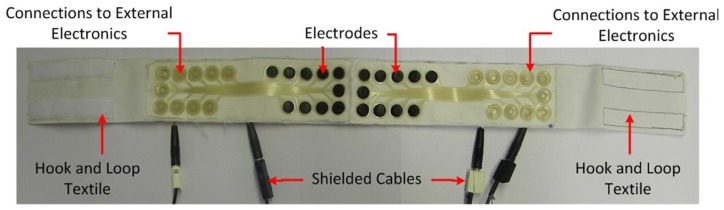
Textile headband for facial EMG.

The researchers developed different bio-potential sensing systems with dry electrodes and conductive ink for signal traces in order to demonstrate that this technology could be used for biomedical application. In particular the application tests were: ECG, facial EMG (Figure 10) and forearm EMG.

#### Related Works

The National Textile Center of the North Carolina State University (Raleigh, NC, USA) is currently working on a project dealing with ‘Printing Electric Circuits on Non-Woven Fabrics’ used to produce a prototype for a physiological monitoring garment that measures ECG, heart-rate, respiration and temperature. In the scope of the project they work together with conductive ink manufacturers. For their experimental investigations and to succeed in producing samples of antennas printed on non-woven textile structures, they used Evolon^®^ by Freudenberg KG (Weinheim, Germany), Tyvek^®^ by DuPont™ (Wilmington, DE, USA) FiberWeb Resolution™ Print Media by BBA FiberWeb™ (Old Hickory, TN, USA) as well as conductive inks made by Precisia LLC (Clark, NJ, USA) and Creative Materials Inc. (Ayer, MA, USA) [62]. Table 3 shows the ink's sheet resistivity.

A group of researchers of Istituto Italiano di Tecnologia—Center for Space Human Robotics, Politecnico di Torino—Applied Science and Technology Department, in collaboration with a spin-off company, Politronica Inkjet Printing S.r.l. (Torino, Italy), developed EMG sensor matrices by inkjet printing a silver nanoparticle-based ink on a polyimide flexible patch. Results indicate excellent behavior with respect to traditional bulk silver systems and a base conductivity of 35% with respect to bulk silver [63].

### 2.5. Conductive Materials as Sensors

Conductive textiles that change their electrical properties as a result of the environmental impact can be used as sensors. Typical examples are textiles that react to deformations such as pressure sensors, stretch sensors and breathing sensors. On the other hand, with smart textiles we have the further possibility to make bio-potential sensors.

#### 2.5.1. Stretch Sensors

Stretch sensors are predominantly used for sensing and monitoring body parameters, as the textile is in contact with the skin over a large body area. This means that monitoring can take place at several locations on the body. For instance, these sensors can be used for determining: heart rate, respiration, movement and pressure blood [64]. A specific structure of textile sensors is that integrating fibers featuring piezo-resistive properties, enabling their use as strain or deformation sensor.

**Figure 11.**
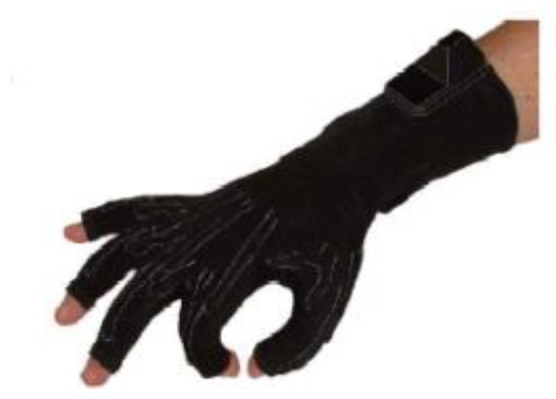
DataGlove™ VRLOGIC with flex sensors.

A first approach to integrate electronics into textile structures was certainly achieved by gloves wired to the computer that allows it to take input from user's hand gestures. Sensors in the glove detect (Figure 11) the wearer's hand movements. Four wires were used for each finger or tube to build up a circuit. The voltages coming out are varying depending on the finger position [65].

##### Related Works

A Flemish consortium of universities and companies, among them the textile department of Ghent University, developed a prototype suit called Intellitex. It is a biomedical suit meant for the long term monitoring of heart rate and respiration of children at the hospital. To measure the ECG, a three-electrode configuration is used. Two measurement electrodes are placed on a horizontal line on the thorax, a third one, acting as a reference (Right Drive Leg, RDL), is placed on the lower part of the abdomen [66].

Philips Research Laboratory (Redhill, UK), developed a stretch sensor integrated into a garment. The stretch sensor, which is produced out of conductive and elastic yarns knitted together, is based on the fact that the electrical resistance changes when stretching the sensing material. Thus, it can be used to control the volume of music or changing the track [67].

#### 2.5.2. Pressure Sensors

Pressure sensors are commonly used either as switches and interfaces with electronic devices or also to monitor vital signs of the user.

Several technologies [68,69], have been developed to manufacture plane pressure sensors. The operating principle is that of changes in piezoelectric resonance frequency with the applied pressure or capacitance variations caused by an elastic foam overlaid with a matrix of conductive threads. For capacitive sensors, a change in parasitic capacitance and resistance can be compensated by the electronics, therefore the wiring has a marginal influence on the sensed signal [9].

**Figure 12.**
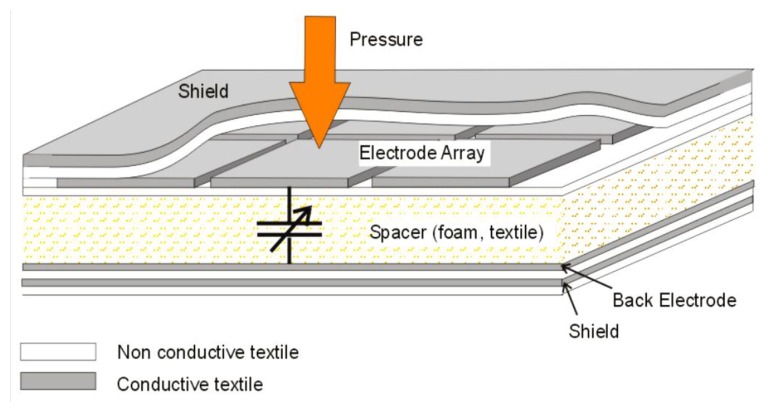
Scheme of sensor with an array of textile capacitors.

The Wearable Computing Lab of ETH Zurich has developed a matrix with several capacitive pressure sensors for integration into a piece of clothing (Figure 12). With this method they are able to measure pressure on a human body and detect muscle activity on the upper arm. Applying this matrix on different body areas, it can provide more details for motion tracking or for the detection of physical state of the muscles [9].

##### Related Works

The British company Eleksen Limited, formerly Electrotextiles (Tunstall, UK), commercializes a soft and flexible textile based sensory fabric under the tradename ElekTex^®^ Smart Fabric Interfaces. It is a combination of conductive fibers and nylon. This combination results into a durable, reasonably priced, washable and even wearable 3D structure [70].

The American-based company Logitech Inc. (Le Lieu, Switzerland) manufactures a soft-touch KeyCaseTM keyboard that can wrap around a personal digital assistant (PDA) for storage and protection. The keyboard is lightweight and made out of textile [71].

**Figure 13.**
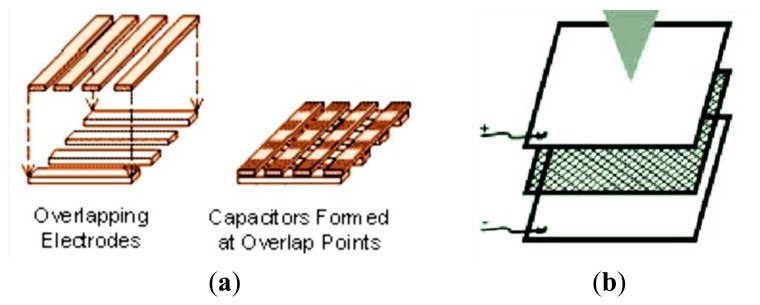
(**a**) Scheme of capacitor sensor developed by the U.S. Company Pressure Profile Systems, Inc. and (**b**) Design for Life Centre at Brunel University in Surrey.

The U.S. company Pressure Profile Systems, Inc. (Los Angeles, CA, USA) designs, develops and manufactures high performance multi-element pressure and tactile sensing systems, called Tactarray and ConTacts (Figure 13a) [72].

The team of the Design for Life Centre at Brunel University in Surrey (UK) has developed a fabric (the Sensory Fabric) that can be used by handicapped children to make themselves understood (Figure 13b). The Sensory Fabric consists of two layers of electrically conductive textile, divided by a layer of non-conductive mesh. When the textile is pressurized, one conductive layer comes into contact with the other, as a result of which an electric stream can flow [73].

The team of the Center for Micro-Bio Robotics of Istituto Italiano di Tecnologia (Pisa, Italy) produced a composite capacitive three-axial sensor based entirely on commercial conductive fabrics, demonstrating its high compliance and stability under manipulation [74].

#### 2.5.3. Electrochemical Sensors

Recent insights into novel fabrication methodologies and electrochemical techniques have resulted in the demonstration of chemical sensors able to augment conventional physical measurements (*i.e.*, heart rate, EEG, ECG, *etc.*) [75]. Recent insights have resulted in the development of a new generation of textile-based chemical sensors that are able to improve conventional physical sensors with more information. Flexible and textile-based screen printed electrochemical sensors may be candidates for non-invasive monitoring, but these devices cannot easily be attached to the body and in particular to the skin.

##### Related Works

**Figure 14.**
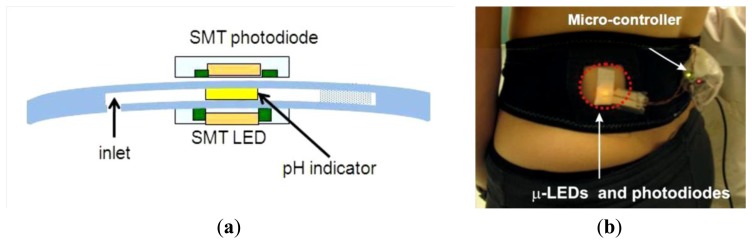
(**a**) Scheme of electrochemical sensor for pH analysis and (**b**) the system application.

The researchers of National Centre for Sensor Research, Dublin City University (Ireland), present examples of wearable chemical sensors that monitor the person and also their environment. In particular the chemical sensor was able to measure and analyze sweat in real-time on the body. They have developed a microchip version of the platform to measure changes in the pH of sweat (Figures 13a and 14a). The color change of the pH sensitive fabric was detected by placing a surface mount (SMT) LED and photodiode module on either side of the chip, aligned with the pH sensitive fabric. The final device (180 μm thick) is flexible and can adapt to the body [76].

#### 2.5.4. Textile Energy Harvesting and Portable Power Supply System

Power supply technologies provide the electrical power for activating the components integrated in the electronic textile; this is still a critical issue in the field of wearable electronics. Although considerable progresses have been seen for wearable electronics, lithium rechargeable batteries, the power sources of the devices, have not kept pace with this progress due to their tenuous mechanical stability, causing them to remain as the limiting elements in the entire technology [77]. For these reasons, the aim is to develop wearable systems capable of accumulating energy dissipated by the body.

The supply of energy by the user's body during everyday actions through leg motions and body heat is also exploited by other research teams. For example, Infineon is currently trying, to recover energy by body movements to feed Mp3 players integrated in a jacket using piezoelectric materials [78]. In the UK the University of Bolton has developed a novel technology that integrates piezoelectric polymer substrate and photovoltaic coating system to create a film or fibre structure that is capable of harvesting energy from nature, including sun, rain, wind, wave and tide [79].

In a project funded by the Engineering and Physical Sciences Research Council (EPSRC), Researchers at the University of Southampton's School of Electronics and Computer Science (ECS) are developing an energy harvesting film in textiles using a rapid printing processes and active printed inks [80,81].

Georgia Tech researchers led by materials-science professor Zhong Lin Wang, have made a flexible fiber coated with zinc oxide nanowires that can convert mechanical energy into electricity. The researchers say the fibers should be able to harvest any kind of vibration or motion for electric current. Gold-plated zinc oxide nanowires, each about 3.5 micrometers tall, are grown on a flexible polymer fiber and these nanowires brush against untreated nanowires, which flex and generate current. Yarn spun from the fibers could lead to fabrics that convert body movements into electric current [82].

Another technology is represented by the solar clothing in which the solar energy is harvested through new generation of flexible solar cells [83,84]. Integration of flexible solar cells into clothing can provide power for portable electronic devices. Photovoltaic is the most advanced way of providing electricity far from any mains supply, although it suffers from the limits of ambient light intensity. Nevertheless the energy demand of portable devices is now low enough that clothing-integrated solar cells are able to power most mobile electronics [85].

##### Related Works

**Figure 15.**
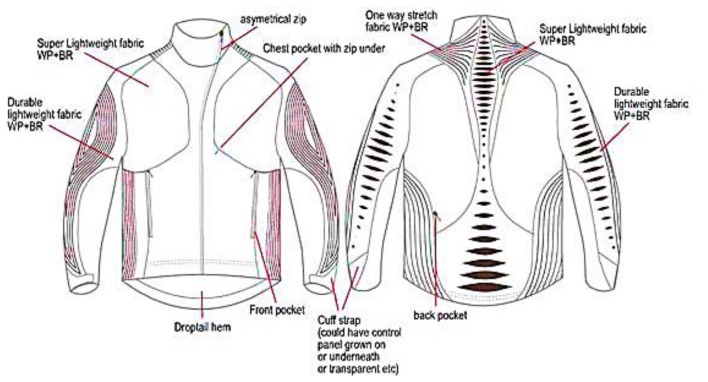
ILLUM jacket layout.

The ILLUM jacket (Figure 15) is based on technologies including printed electroluminescent ink and printed photovoltaic technology. The functional parts are placed outside the jacket and into several ergonomic panels, while at the front and the photovoltaic source at the shoulders and top of the back [86].

Thermotron of UNITIKA (Osaka, Japan) is a particular fabric able to converts sun light into thermal energy while storing heat without wasting it. Inside the Thermotron there are microparticles of zirconium carbide which allow the fabric to absorb and filter sunlight. The inner layer of the fabric withholds the heat generated and prevents it from becoming lost, thus providing a salutary effect on the human body [87].

An example of a battery capable of providing electrical power for interactive electronic textiles was recently developed by a German research team led by The Fraunhofer Institute for Reliability and Micro-integration (FhG-IZM, Berlin, Germany). This research team developed a small battery that can be printed on a substrate and fabricated at high production speeds in button-sized or coin-type format at a cost below one USD. The battery is fabricated by screen printing a thick layer of a silver-oxide based paste, then applying a thin sealing layer. The final result is a textile substrate with a printed 120 μm thick AgO-ZN battery. These batteries can be printed on a variety of substrates [88].

### 2.6. Planar Fashionable Circuit Board (P-FCB)

The P-FCB is one of the new technologies allowing implementation of a circuit board on a plain fabric patch for wearable electronics applications. It features a soft and flexible impression just as normal clothes.

**Figure 16.**
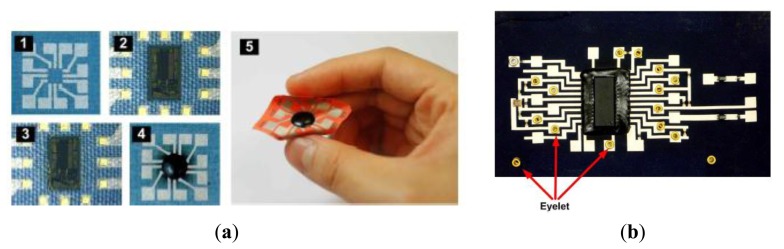
(**a**) Single-layered P-FCB system manufacturing process; (**b**) Implementation of multi-layer connection using eyelet.

The P-FCB substrate is fabricated using woven fabrics. The planar electrodes are deposited on the fabric patch directly by silk screening of conducting epoxy or by gold sputtering. First, the circuit board is silkscreen printed on the fabric patch. Then the IC is placed on the fabric and wire-bonded to the patterned electrodes. Finally, the IC is molded with non-conductive epoxy (Figure 16a) [89].

With these techniques, KAIST researchers also developed a multilayer circuit (Figure 16b). The previous research highlight how electrical and mechanical characteristics and valuable system design parameters are obtained, such as maximum power consumption, maximum current density, crosstalk between neighboring two lines, and durability [90].

### 2.7. Wearable Antenna

Thanks to the rapid progress on the fabrication of conductive textiles, a significant development of wearable antennas has begun, exploiting new flexible and conformable smart structures [91].

An antenna is essential, if the purpose is to develop a wearable and autonomous system. It allows one to transfer information from the sensors hosted inside the garment to a control unit or to monitor other electronic parameters.

A wearable antenna is thus the bond that integrates clothes into the communication system, making electronic devices less obtrusive. To achieve good results, wearable antennas have to be thin, lightweight, low maintenance, robust, inexpensive and easily integrated in radio frequency (RF) circuits. Planar structures, flexible conductive and dielectric materials are specific requirements for wearable antennas [92,93].

Several properties of the materials influence the behavior of the antenna. For instance, the bandwidth and the efficiency of a planar microstrip antenna are mainly determined by the permittivity and the thickness of the substrate [94,95]

In general, textiles present a very low dielectric constant that reduces the surface wave losses and increases the impedance bandwidth of the antenna. Therefore it is important to know how these characteristics influence the behavior of the antenna in order to minimize unwanted effects.

Another issue regards the movement of the body that can deform the spatial geometry of the antenna and affect its performance. When the textile fabric adapts to the surface topology it bends and deforms, causing changes to its electromagnetic properties and thus influencing the antenna performance [96]. Thus, a wearable FM antenna should be designed so as to be wider than the FM broadcast band (about 81–130 MHz) not to suffer from the detuning caused by the human body [97].

To summarize, the guidelines for a correct antenna project are shown below:

- choosing the correct positioning of the textile antenna [98,99];
- the textile antenna must be made with an accurate thickness stacking the different fabrics [99];
- the geometrical dimensions of the patch must remain stable [100];
- the connections between the layers must not affect the electrical properties and the connections with other parts of “e-garments” have to be stable and robust [101–103].

If some of these points are not followed, undesired effects may occur to the functioning of the device.

#### Related Works

Researchers at Katholieke Universiteit Leuven and Universiti Malaysia Perlis were the first to develop a fully textile waveguide antenna using a material inspired unit cell that is also used in composite right/left-handed transmission lines. The antenna is compact, robust and can be used for 2.45 and 5.4 GHz dual-band WLAN applications [104].

Patria (Halli, Finland) is a company with expertise in textile antenna design. It develops textile antennas composed by conventional or industrial fabrics, and typically conductive antenna parts are made out of modern conductive fibers [105].

Textiles RFID is a particular solution of antenna. In this sector TexTrace AG (Frick, Switzerland) provides the manufacturing line as well as the components for industrial in-house production of woven RFID labels. Integrate RFID and the label will provide added value from garment manufacturing through logistics to sales and after-sales management [106].

### 2.8. Stretchable Interconnection

Deformable electronic circuits, in particular in bio-medical application, are needed. E-textiles are the candidate technology for this scope but an important aspect regards the interconnections between components and devices. In most cases the approach used is to develop sinuous electroplated metallic wires in a stretchable substrate material [107].

**Figure 17.**
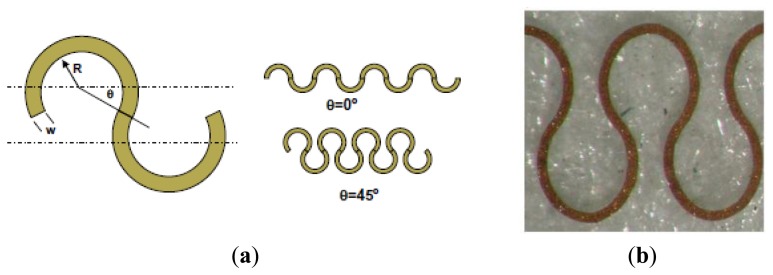
(**a**) Notable measures of a horseshoe design: inner radius (*R*), joining angle (*θ*) and width of the metal track (*w*); (**b**) Horseshoe metal interconnects embedded into a Sylgard 186 PDMS matrix.

Figure 17 presents a stretchable interconnection structure. This configuration, called “horseshoe-shape”, is able to accommodate large deformation in response to a mechanical stress, preserving the electrical properties [108].

Indeed, with high-frequency signal the interconnection have to be stretchable and therefore the substrate and the conductors have to be stretchable as well. Usually, a polymeric material is chosen as encapsulating substrate, because it can be stretched and also because it may be bio-compatible [109].

Interconnections made with these techniques coupled with conductive yarns can go beyond actual issues regarding the lack of robustness in e-textiles circuit.

## 3. Discussion

The methods presented in the previous section are quite different and each one has very specific feature such as conductivity, flexibility, biocompatibility, mechanical resistance and washability. However, only a few approaches are able to satisfy all these requirements at the same time. The section below has the aim to clarify some features about the materials and methods.

### 3.1. Conductive Wires

Especially for clothing, tactile properties such as stretch, recovery, drape, shear and handle are quite important. For this reason the fibers that are used should be fine and fabrics should have a low weight per unit area (not more than 300 g/m^2^). These demands are inconsistent with the materials and geometries that are needed for a reasonable electrical conductivity, because the incorporation of elements such as metal wires within textiles increases stiffness and reduces elasticity [110].

Often in literature no distinction is made between metal wires and metal fibers. However, the company Sprint Metal (Hemer, Germany) defined metal fibers and wires according to their diameter. While a fine wire has a diameter between 30 μm to 1.4 mm, a metal fiber possesses a diameter of 2 to 40 μm [111].

The advantages of metal fibers are their strength, composition, biological inertness and ready availability in textile form at low costs. Due to its inertness it is not sensitive to washing or sweating.

However, they cannot provide uniform heating and their brittle characteristics can damage spinning machinery over time. Additionally, they are heavier than most textile fibers making homogeneous blends difficult to produce [112].

Using the ’conductive thread’ approach, no additional step after manufacturing of the fabric is required to establish conductivity. The conductivity of these conductive threads lies in the range of 10–500 Ω/m [113].

For instance, Figure 4a shows the copper wire twisted around the yarn and their locations are not precisely defined within the fabric because of their helical path around the yarn [114]. The conductance of a wire in the fabric features about 17.8 Ω/m.

### 3.2. Treated Coatings

The advantage of coatings is that they are suitable for many fibers types and they produce good conductivity without significantly altering existing key substrate properties such as density, flexibility and handle. Nevertheless adhesion between the metal and the fibers as well as corrosion resistance can lead to problems.

It is possible to make a conductive fabric through a coating treatment even though the main applications for these textiles are protection against static electricity charge and electromagnetic interference (EMI). The surface resistivity (Rs) of normal textiles goes from more than 10^5^ Ω/sq down to antistatically finished textiles and conductive textiles for EMI and heating applications, where Rs can be lower than 100 Ω/sq. It is usual to incorporate or impregnate organic antistatic agents [98].

**Figure 18.**
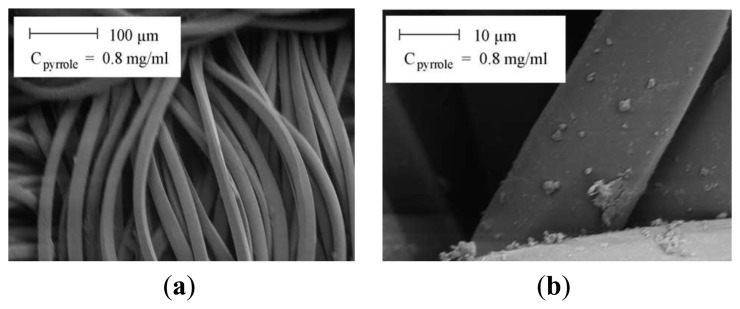
SEM image of the polypyrrole coated textile (C_pyrrole_ = 0.8 mg/mL), (**a**) 100 μm scale and (**b**) 10 μm scale.

At present the most important ways of producing conductive fibers are:

- incorporation of conductive fillers (carbon black, metal wires, graphite and metal powder or flakes of Al, Cu, Ni, Ag).
- blended fabrics using conductive metallic or polymeric fibers (such as polypyrrole, polyaniline) (Figures 16b and 18a) [115].

However, the surface resistance of polypyrrole-coated (C_pyrrole_) polyester fabrics is another method to functionalize the textiles. Rs decreased marginally from 106 Ω/sq to 103 Ω/sq with increasing pyrrole concentration within 0.2 mg/mL until the concentration reached a value of about 0.4 mg/mL, above which the rate of decrease diminished. The decrease in surface resistance is due to the increase in the thickness of the conducting polymer layer [116].

The main advantage of functionalized yarns is that the textile properties of the yarns are preserved, in particular they remain flexible.

### 3.3. Conductive Fabric PETEX

**Figure 19.**
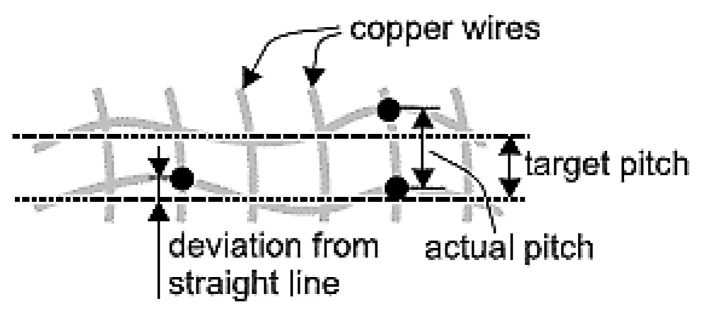
Misalignment of the contact points.

Normally, a fabric made with conductive fibers has a structure according to Figure 19. If the aim is to exploit the conductive fibers to create an electrical circuit, we must take into account the irregular shape of the fabric. It is not possible to obtain a geometric repeatability of the position of the contact points between the conductive fibers.

The solution adopted by the ETH researchers is to develop a synthetic fabric and it consists of woven polyester monofilament yarn (PET) with diameter 42 μm and copper alloy wires with diameter 50 ± 8 μm (AWG461). Each copper wire itself is coated with a polyurethane varnish as electrical insulation. The wires in warp direction possess a higher tensile strength compared to the wires in weft direction. These differences support a reliable manufacturing process and minimize breakage.

**Figure 20.**
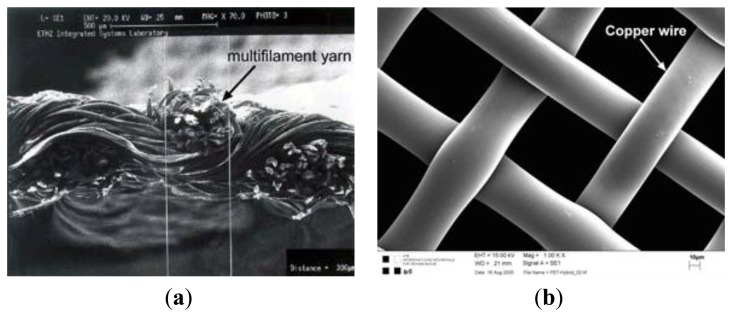
SEM image of fabric with copper fiber (**a**) and PETEX (**b**).

Figures 18b and 20a below, show the differences between a normal electronic textile structure and PETEX [47].

### 3.4. MIT CAD Embroidery

This technique allows precisely specifying the circuit layout and stitching pattern in a computer-aided design (CAD) environment, from which any number of articles can be sewn under machine control. This process also allows control and integration of yarns with different electrical properties, for instance, different resistances. Embroidery offers advantages over knitting or weaving. Conductive thread and yarn embroidery can be accomplished on single or multiple layers of fabric or can be applied on various types of textile and apparel products in one step [39].

### 3.5. Conductive Inks

**Figure 21.**
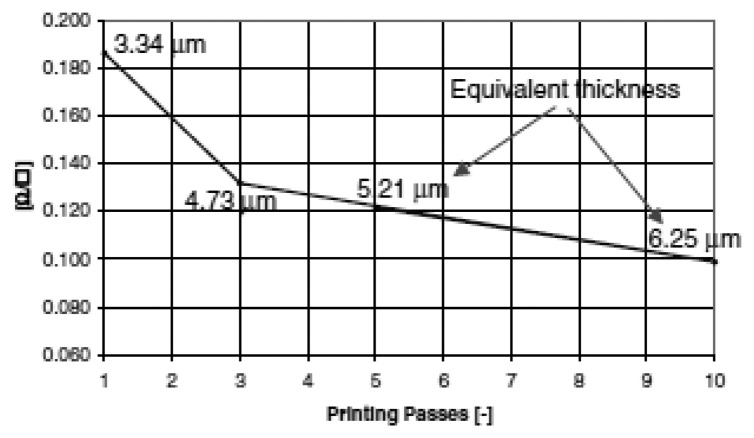
Sheet resistance *vs*. printing passes.

Direct printing of conductive tracks on a fabric is a versatile technique but sometimes shows its limits. Most conductive inks and pastes are based on silver filler and suffer from brittleness. During printing, one of the limits of the silver paste is the thickness. For instance, several passes are needed to achieve a layer thick 40 μm and the electrical resistance depends on the number of passes applied (Figure 21).

**Figure 22.**
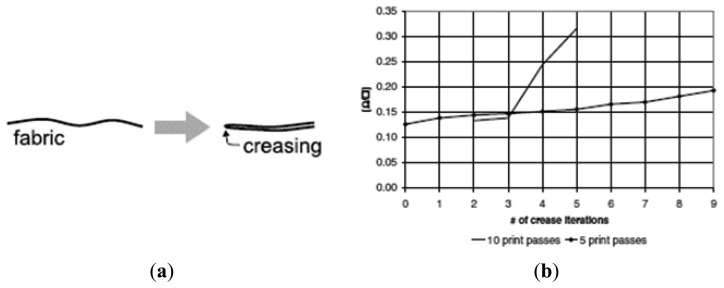
(**a**) Creasing of the fabric with printed transmission lines; (**b**) DC resistance changes *vs.* number of creasing iterations.

Another problem is the mechanical resistance. If you create a condition as shown in Figure 22a, the risk is to have a break in the conductivity. In particular the electrical conductivity changes both for the number of passes and for the number of creasing interactions (Figure 22b) [47].

The researchers of North Carolina Institute, developed a new method to control the durability of the printed circuits, which includes coating of the printed lines. The viscosity of the ink dictated the performance of the printed media during washing trials.

**Figure 23.**
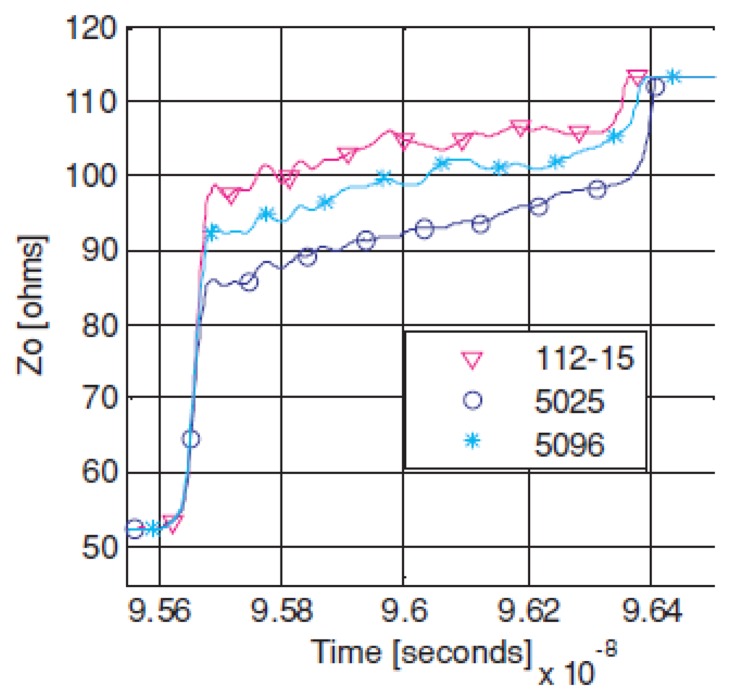
Evolon impedence profile before five washes. The three lines show the comparison between different conductive inks (Dupont 5025, Dupont 5096, Creative Materials 112-15).

The results showed that higher viscosity inks stay on the surface, resulting in better short-term results. (Figure 23) The ink used was produced by Creative Materials and showed rather a smooth and lower impedance profile than other commercial conductive inks. This ink is based on silver particles and the lines printed on Evolon^®^ show 42 times higher resistivity than standard PCBs (0.50 mΩ/m). However, Evolon^®^ like structures with the highest viscosity ink represent the best solution in terms of durability and conductivity also after washing. This substrate-ink combination protected by a TPU shows the best compromise to become a low-cost electronic textile solution for wearable computing interconnects [117].

### 3.6. Stretchable Sensors

Usually, textile materials composed of fibers form complex networks of conducting parts that make multiple contacts. During deformation a number of mechanisms take place:

- the number of contact points changes;
- fibers are extended;
- fiber cross-section is decreased.

These factors listed above may compromise the stretch sensors qualities [118]. Polymers are interesting when developing strain sensors with large strain [1]. The stretch sensor developed by Mattmann *et al.* [119] consists of a mixture of 50 wt-% thermoplastic elastomer (TPE) and 50 wt-% carbon black particles and is fiber-shaped with a diameter of 0.315 mm. This sensor configuration was characterized using a strain tester and measuring the resistance (extension-retraction cycles): it showed a linear resistance response to strain, a small hysteresis, no ageing effects and a small dependence on the strain velocity. The attachment of the sensor to the textile is realized using a silicone film.

**Figure 24.**
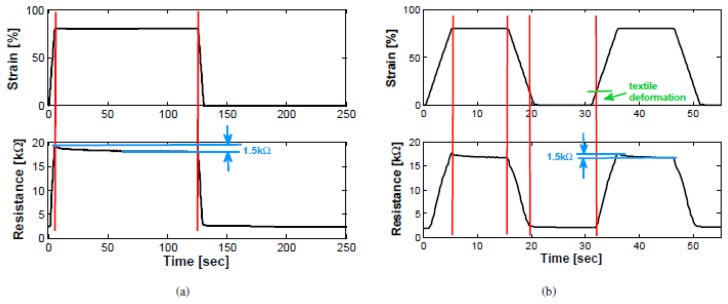
(**a**) Typical response of sensor to a given strain (sensor length 2 cm) waiting time 2 min; (**b**) waiting time 10 s.

**Figure 25.**
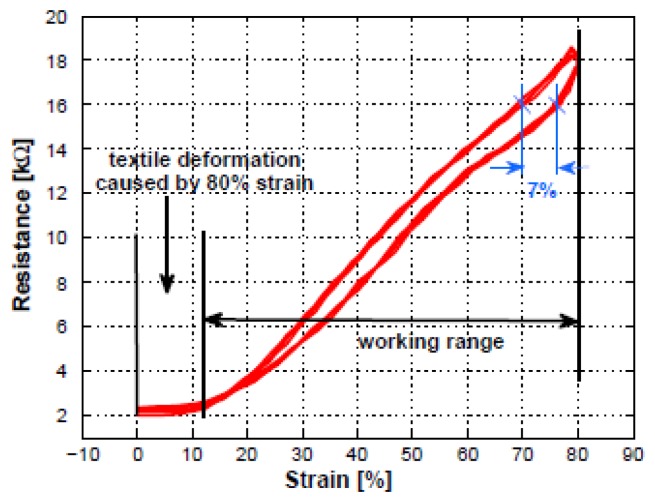
Typical response of sensor to a given strain.

The graphs (Figure 24) below show the behavior of the sensor and the typical resistance *vs.* time plot. The sensor was cycled between 0% and 80% strain at a speed of 200 mm/min and waiting times at minimal and maximal strain of 2 min. In the lower plot the measured resistance is shown which varying between 2 kΩ and 19 kΩ. Instead, Figure 25 shows a linear rise in resistance when applying strain and only a small hysteresis.

Commercially available strain gauges reach a higher than 80% linearity but at a reduced working range of less than 1%. The sensor presented above has a high sensitivity of 1.25 kΩ/mm with a stable sensor properties while in continuous usage. Furthermore the sensor is washable.

A similar approach is that used by Shyr *et al.*, in which they developed three different geometries of a strain-resistance sensors using Lycra fiber and carbon coated polymide fiber: flat, tubular and belt webbings. It was found that tensile hysteresis and contact resistance significantly influence the tensile elasticity and the resistance sensitivity of the webbings, respectively. Their results showed that all the webbings had a good linear elasticity relationship between tensile load and strain when stretched with the aim to reduce the tensile hysteresis, decreasing the friction among the conductive yarns [118]. The stability of resistance sensitivity and the elasticity are two relevant properties for e-textiles when used as a strain-resistance sensor.

### 3.7. Pressure Sensors

**Figure 26.**
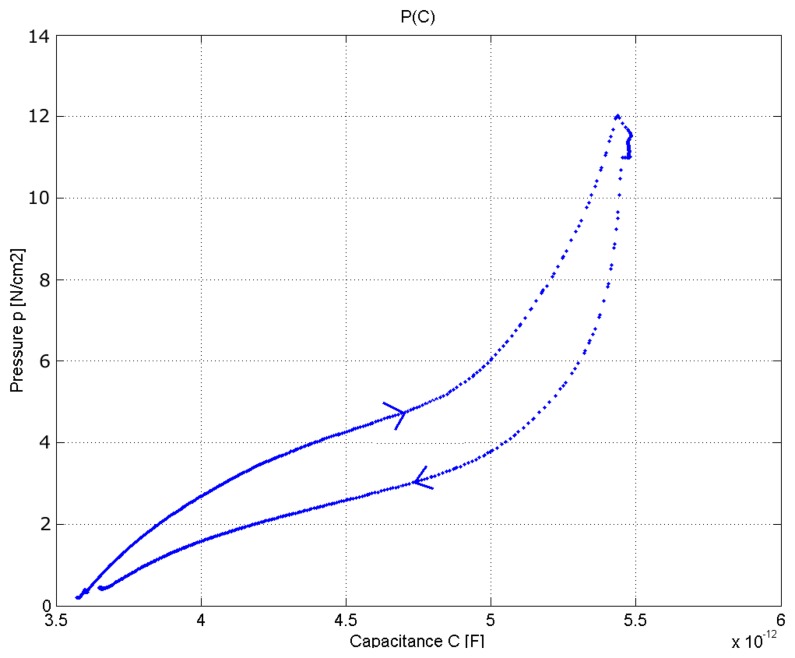
Hysteresis of a singular pressure electrode.

The pressure sensor developed by Meyer *et al.* [9] has a spatial resolution of 2 × 2 cm and an average error below 4% within the measurement range 0 to 10 N/cm^2^. It is a pressure sensor based on capacitive behavior. The measured capacitance of a single electrode of the prototype with the textile spacer varies from 3.5 pF uncompressed to 5.8 pF at a pressure of 5 N/cm^2^ for the textile spacer with 6 mm thickness. Figure 26 shows the capacitance *vs.* pressure with a hysteresis up to 30%. The high value of hysteresis is due to the dielectric material interposed between the two conductive faces capacitor.

An ideal pressure sensor must ensure repeatable measures over time with a low hysteresis value. Therefore, the textile pressure sensors are applicable in areas where high accuracy is not required.

### 3.8. Electrochemical Sensors

One of the common disadvantages of chemical/biochemical sensors is the need to calibrate the response from time to time due to either small changes in the activity of the sensor itself and/or the contamination of the sensor surface during operation. This has seriously limited the application of these sensors in continuous monitoring scenarios for long time. The major obstacles still remain, preventing the realization of practical, robust wearable sensors suitable for routine use by the general population.

**Figure 27.**
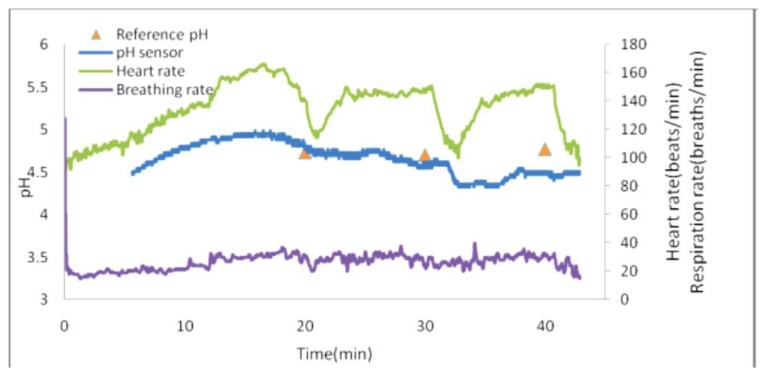
Wearable sweat sensors performance.

Among these limitations, we have the lack of appropriate methods to seamlessly integrate electronics and wireless transmitters into the sensor package. The graph below (Figure 27) shows the performance of the wearable sweat sensor during an exercise trial. Reference pH measurements are taken along with heart rate and breathing rate data. The analysis were taken by placing the electrode on a reference fabric patch, as performed in previous studies [120].

### 3.9. Power Supply

In the following Table 4, an overview of different possible energy sources and the amount of energy that they can generate is given [120,121].

From these data, we can assume that the energy harvesting coming from our body is very small although it is always present. If the wearable technologies will take advantage of these energy sources, they must feature a low electrical consumption and a high efficiency harvesting system.

### 3.10. Planar Fashionable Circuit Board (P-FCB)

The measured sheet resistance of the conducting film on the P-FCB is 44 mΩ/m, as determined by the four-point probe method. Although no noticeable degradation has been observed, even after 50 laundry cycles, more durability examinations will be required with an automatic testing system.

**Figure 28.**
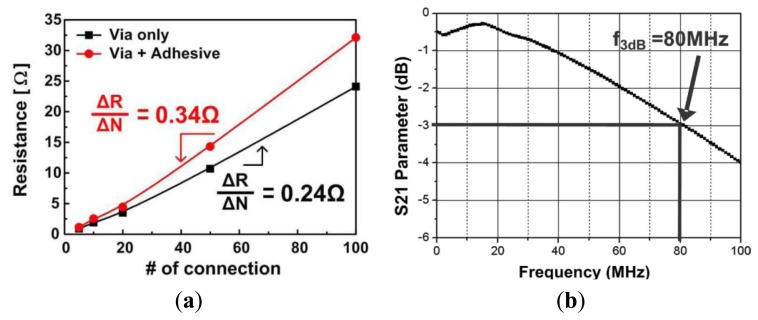
(**a**) Measurement result of the proposed via and conductive adhesive resistance; (**b**) Bandwidth of a P-FCB transmission line (15 cm long, 1 mm wide).

The resistance measurement results of the proposed via and conductive adhesive and bandwidth of a P-FCB transmission line 15 cm long and 1mm wide are shown in Figures 26b and 28a. Its bandwidth is 80 MHz, and this is sufficient to deal with bio-signal processing. The analysis were performed with a vector network analyzer [122]. To get an average value, as many as 100 connections were formed and measured. The resistances of the proposed via and conductive adhesive measured 0.24 W and 0.34 W, respectively [123].

Mechanical strength of the P-FCB was tested through washing tests; after 50+ cycles, the electrical characteristics change was negligible [123]. As a result, the monitoring shirt can be easily made at low cost, and the wearability and flexibility are improved.

### 3.11. Wearable Antenna

Antennas for wearable applications need to be comfortable to wear and traditional technology with rigid substrates is not well suited to this.

In this sector the dielectric properties of the material that make up the antenna are more relevant. A wrong choice of material would compromise the performance of the device.

The constitutive parameter of dielectrics is the permittivity, ε, a complex value parameter and it usually expressed by Equation (1) below:

(1) $${ɛ}_{r}:ɛ={ɛ}_{0}\cdot {ɛ}_{r}={ɛ}_{0}\cdot ({ɛ}_{r}^{\prime }-\text{j}\cdot {ɛ}_{r}^{\prime \prime })$$

where ε_0_ is the permittivity of vacuum, 8.854 × 10^−12^ F/m [124]. The dielectric properties are a function of different physical parameters: temperature, frequency, surface roughness, moisture content, purity and homogeneity of the material.

Table 5 below shows the dielectric constant ***ε*** *′_r_* and the ratio of the imaginary part to the real part, also known as *tan δ* = ***ε****″_r_/****ε****′r* [94].

The low dielectric constant (between 1 and 2) reduces the surface wave losses that are tied to guided wave propagation within the substrate. Therefore, lowering the dielectric constant increases spatial waves and hence increases the impedance bandwidth of the antenna [125].

In addition, the moisture can affect the dielectric behavior of textiles. In particular, when water is absorbed by the fibres, an increase of the dielectric constant and loss tangent occurs. A textile cover or superficial treatment may then provide a sufficient protection from the humidity and varying climatic conditions [100].

Researchers of Tampere University of Technology, in collaboration with Patria, developed a robust textile antenna able to operate in different environmental conditions. They demonstrated that different functional textile layers can improve and optimize the antenna performance. In this occasion they succeeded to protect the antenna against moisture and others environmental factors [126].

The thickness of the dielectric material changes the bandwidth of the planar antenna. It is necessary to find a compromise between efficiency and bandwidth, well as to influences the geometric sizing of the antenna [125].

For the antenna design, the relevant parameter is the conductivity of the fabric (σ), which unit is Siemens per meter (S/m). It is expressed by this Equation (2):

(2) $$\sigma =1/(\rho_{s}\cdot \text{t})$$

where *ρ*_S_ is the surface resistivity and t is the thickness of the fabric. An issue regarding the conducibility of conductive fabrics for antenna is the homogeneity of σ value; In fact, there are several discontinuities inside the fabric and these affect the current flow [127].

Another aspect that influences the conductivity is the structure of the fabric. The current flow depends on the type of textile structure used and to minimize the conductive losses it is better to align the conductive paths with the current direction [98].

Finally, a correct choice of materials combined with textile structures can improve the performance of the textile antenna equaling the features guaranteed by traditional antennas.

### 3.12. Stretchable Interconnections

**Figure 29.**
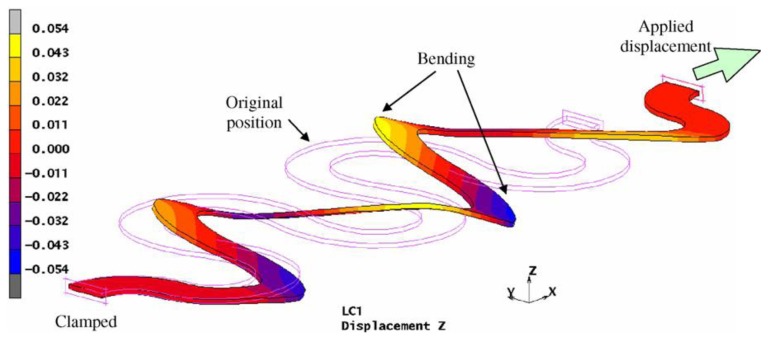
FEM simulation of the “horseshoe-shape”.

Stretchability of the electrical conductors is a requirement that can be satisfied with different designs. The design optimization is done using FEM modeling (Figure 29) and other tests. Gonzalez *et al.* evaluated the “horseshoe-shape” to be the optimal solution for this purpose [108]. In this way the damage in the conductor is significantly decreased with a larger deformability. They demonstrated that in case of multiple and narrow metallisations the stretchability of the circuit reaches 100% of the deformability.

Hu *et al.* used a different approach to obtain a stretchable conductive textile. With a simple “dipping and drying” process using single-walled carbon nanotube (SWNT) ink, they produced highly conductive textiles with conductivities of 125 S·cm^−1^ and sheet resistances less than 1 Ω/sq. In this way, such conductive textiles preserve the same stretchability of the normal fabric. Indeed, the porous structure of the textiles facilitates accessibility from any electrolyte and such porous and stretchable conductors find a wide range of applications in e-textiles field [128].

## 4. Conclusion/Outlook

Textiles represent an attractive class of substrates for realizing wearable bio-sensors. Electronic textiles, or smart textiles, describe the convergence of electronics and textiles into fabrics which are able to sense, compute, communicate and actuate. As many different electronic systems can be connected to any clothing, a wearable system becomes more versatile, and the user can change its look depending on environmental changes and individual preference.

The vision of wearable computing describes future electronic systems as an integral part of our everyday clothing serving as intelligent personal assistants. Therefore, such wearable sensors must maintain their sensing capabilities under the demands of normal wear, which can impose severe mechanical deformation of the underlying garment/substrate.

One promising approach to reduce the rigidity of electronic textiles and enhance its wearability is to replace PCBs by flexible electronics.

In this review we wanted to explain how it is possible to develop a Smart Textile. Some methods show advantages with respect to others, but in our opinion and in according to the consulting company Smart Garment People (Lancashire, UK), while some manufacturers are very experienced with electronics and others with textiles, very few do both well.

Current advances in textile technologies, new materials, nanotechnology and miniaturized electronics are making wearable systems more feasible but the final key factor for user acceptance of wearable devices is the fit comfort. We are convinced that this goal can only be achieved by addressing mechanical resistance, and durability of the materials in what is recognized to be a harsh environment for electronics: the human body and society.

Finally, we consider relevant that the development of smart textiles requires a multidisciplinary approach in which knowledge of circuit design, smart materials, micro-electronics and chemistry are fundamentally integrated with a deep understanding of textile fabrication.

## Figures and Tables

**Table 1.** Smart Textiles project—EU FP6 and FP7.

**Project Title**	**Description**
WEALTHY Sept 2002–Feb 2005 http://www.wealthy-ist.com/	Pioneering research to develop and test comfortable smart fabrics for biological monitoring–ECG and respiration.
MyHeart Jan 2004–Oct 2007 http://www.hitech-projects.com/euprojects/myheart/	Development of Intelligent Biomedical Garments for monitoring, diagnosing and treatment of medical conditions
BIOTEX Oct 2005–Feb 2008 http://www.biotex-eu.com/	Sought to develop biochemical-sensing techniques that could integrate into textiles. Project aimed to develop textile sensing patches to target bodily fluid sensing.
PROETEX Feb 2006–Jan 2010 http://www.proetex.org/	Developed smart wearable garments for emergency disaster intervention personnel to improve safety, coordination, and efficiency.
STELLA Feb 2006–Jan 2010 http://www.stella-project.de/	Sought to develop flexible and stretchable textile substrates with electrical interconnects.
OFSETH Mar 2003–Jun 2009 http://www.ofseth.org/	Focused on how silica and polymer optical fibers can be used for sensing vital parameters while being compatible with a textile manufacturing process.
CONTEXT Jan 2006–Jun 2008 http://www.context-project.org/	Focused on development of contactless sensors in textiles for monitoring ECG and EMG.
WearIT@Work Jun 2004–Nov 2008 http://www.wearitatwork.com/	Aimed to prove the applicability of computer systems integrated to clothes, creating wearable interfaces for various industrial environments.
MICROFLEX May 2008–May 2012 http://microflex.ecs.soton.ac.uk/	Development of flexible materials in the form of high added value smart fabrics/textiles which are able to sense stimuli and react or adapt to them in a predetermined way.
DEPHOTEX Nov 2008–Oct 2011 http://www.dephotex.com/	Research and development of flexible photovoltaic textiles based on novel fibers
PLACE-it Feb 2010–Jun 2013 http://www.place-it-project.eu/	Development of a technology platform for lightweight, thin and conformable opto-electronic systems interconnect technology

**Table 2.** Electrical properties of metal monofilaments fibers.

**Metal**	**Electrical Properties**				

	**Conductivity [S·m/mm^2^)**	**Resistivity [Ω·mm^2^/m]**	**Thermal Coefficient of Resistance [10^−6^ K^−1^]**		

			**Min**	**Typ**	**Max**
Cu	58.5	0.0171	3900	3930	4000
Cu/Ag	58.5	0.0171	3900	4100	4300
Ag 99%	62.5	0.0160	3800	3950	4100
Ms * 70	16.0	0.0625	1400	1500	1600
Ms/Ag	16.0	0.0625	1400	1500	1600
AgCu	57.5	0.0174	3800	3950	4100
Bronze	7.5	0.1333	600	650	700
Steel 304	1.4	0.7300		1020	
Steel 316L	1.3	0.7500		1020	

* German Milbe denomination, where “Ms” is accompanied by a number stating the composition in %Cu with respect to a Zn complement to 100%.

**Table 3.** Electrical properties of inks.

**Inks**	**Sheet Resistivity (Ω/sq/mil)**
CMI 112-15	0.010
DuPont 5025	0.010–0.012
DuPont 5096	<0.010

**Table 4.** Comparison of different energy source and their energy capability.

**Energy Source**	**Available Amount of Energy**	**Remarks**
Primary batteries (Li)	400 Wh/kg, 200 Wh/L	
Secondary batteries (Li-Ion)	75 Wh/kg, 200 Wh/L	Lifetime: 2000 cycles
Si solar cells	20 W/m^2^	Light source needed
Recovery of body heat	0.01 W/m^2^	
Power harvesting from breathing	0.4 W	Uncomfortable for wearer
Power harvesting from walking	0.25 W	Continuous walking necessary
Microcombustion	10–50 W/cm^3^	Fuel needed

**Table 5.** Dielectric Properties of normal fabrics.

**Nonconductive Fabric**	***ε'_r_***	**tan δ**
Condura ^®^	1.90	0.0098
Cotton	1.60	0.0400
Polyester	1.90	0.0045
Quartzel ^®^	1.95	0.0004
Cordura/Lycra^®^	1.50	0.0093
